# Engagement with eHealth Self-Monitoring in a Primary Care-Based Weight Management Intervention

**DOI:** 10.1371/journal.pone.0140455

**Published:** 2015-10-15

**Authors:** Kathleen Y. Wolin, Dori M. Steinberg, Ilana B. Lane, Sandy Askew, Mary L. Greaney, Graham A. Colditz, Gary G. Bennett

**Affiliations:** 1 Coeus Health, Chicago, IL, United States of America; 2 Duke University, Durham, NC, United States of America; 3 University of Rhode Island, Kingston, RI, United States of America; 4 Washington University in St. Louis, St. Louis, MO, United States of America; Karolinska Institutet, SWEDEN

## Abstract

**Background:**

While eHealth approaches hold promise for improving the reach and cost-effectiveness of behavior change interventions, they have been challenged by declining participant engagement over time, particularly for self-monitoring behaviors. These are significant concerns in the context of chronic disease prevention and management where durable effects are important for driving meaningful changes.

**Purpose:**

“Be Fit, Be Well” was an eHealth weight loss intervention that allowed participants to self-select a self-monitoring modality (web or interactive voice response (IVR)). Participants could change their modality. As such, this study provides a unique opportunity to examine the effects of intervention modality choice and changing modalities on intervention engagement and outcomes.

**Methods:**

Intervention participants, who were recruited from community health centers, (n = 180) were expected to self-monitor health behaviors weekly over the course of the 24-month intervention. We examined trends in intervention engagement by modality (web, IVR, or changed modality) among participants in the intervention arm.

**Results:**

The majority (61%) of participants chose IVR self-monitoring, while 39% chose web. 56% of those who selected web monitoring changed to IVR during the study versus no change in those who initially selected IVR. Self-monitoring declined in both modalities, but completion rates were higher in those who selected IVR. There were no associations between self-monitoring modality and weight or blood pressure outcomes.

**Conclusions:**

This is the first study to compare web and IVR self-monitoring in an eHealth intervention where participants could select and change their self-monitoring modality. IVR shows promise for achieving consistent engagement.

## Introduction

There is a need for weight loss interventions that can effectively reach medically vulnerable populations and that have the possibility of being cost-effective and sustainable. eHealth interventions (health interventions delivered via the Internet or telephone) have the potential to address each of these goals, and have become quite prevalent across health outcomes in recent years [1]. Several recent reviews note that eHealth intervention approaches produce clinically significant changes in health risk factors and weight [1–3]. However, the degree of weight change has typically been less than what has been observed for in-person interventions conducted at the individual or group level [1, 4, 5]. It has been suggested that this might result from the lower participant utilization and declining engagement over time in eHealth interventions [6]. Several recent reviews have reported attrition rates of 40–50% or higher in these types of interventions as well as a stark decline in eHealth intervention engagement after the first few weeks of intervention [1, 3].

Across intervention modalities (e.g., web, phone, email), engagement is reliably associated with better weight loss outcomes [7]. While eHealth interventions hold great promise, they often have lower than expected engagement [1, 3]. Intervention engagement is typically measured by focusing on adherence to self-monitoring (historically through logs in paper/journal format) in behavioral interventions for weight loss, weight maintenance, diet changes and increasing physical activity [7, 8]. Emerging evidence suggests that simpler tools, such as the use of web-based components in eHealth interventions [6, 9], may be equally effective [3, 10–13]. Increasing engagement is a priority as eHealth interventions proliferate and obesity rates remain unacceptability high, particularly among socioeconomically disadvantaged and racial/ethnic minority groups. Unfortunately, few interventions have been tested in the populations at greatest need. In fact, most eHealth interventions have been tested in samples consisting of highly motivated, socioeconomically advantaged participants[14, 15], which limits their generalizability.

Historically, most studies offer a single type of self-monitoring or require participants to choose one modality at baseline. These modalities vary in their context, accessibility, portability, ease of use and necessary health and technology literacy. We hypothesized that greater flexibility in choosing an eHealth modality might increase engagement in self-monitoring in an eHealth intervention because there might be variation among participants regarding their experience with, and preference for, the modalities. Recognizing that over the course of a long intervention, participant access to and comfort with technologies might change, we were interested in both giving people an initial choice of monitoring modality and the option to change modalities. Therefore, we examined this in “Be Fit, Be Well” (BFBW), a 24-month trial of an eHealth weight loss intervention conducted in the primary care setting among medically vulnerable obese patients with hypertension. Intervention participants could choose between two modalities for receiving the intervention: a comprehensive study website for content and self-monitoring or printed materials plus IVR (interactive voice response) self-monitoring [11]. Both intervention modes were designed to assist study participants in achieving weight loss by targeting and changing specific tailored behavioral goals, which they were encouraged to self-monitor on a weekly basis.

The present study investigates BFBW participant engagement in greater specificity with specific attention to differences by intervention modality and among those who changed their means of engaging with the study. We (1) describe patterns of engagement, (2) examine selected correlates of participant engagement in the intervention, and (3) explore the association of participation engagement with weight loss and blood pressure outcomes.

## Subjects and Methods

### Design and participants

The design and primary results of BFBW have previously been published [11, 14]. Briefly, BFBW was implemented in three community health centers (CHC) in Boston, Massachusetts. Patient eligibility requirements included having a body mass index (BMI) of 30–50 kg/m^2^ (and weighing less than 181.8 kg.), receiving pharmacologic treatment for hypertension, age of at least 21 years, being a patient at the participating CHC, fluency in English or Spanish, being willing to change diet, physical activity and weight and providing written informed consent. Participants completed an evaluation visit every 6 months during which trained research staff measured their weight, height and blood pressure and participants took a self-administered computer-based survey. Following the baseline assessment, study participants (n = 365) were randomized to receive either usual care (N = 185) or the BFBW intervention (N = 180) [14]. Of the original sample, 314 men and women (86% of the original sample) completed the 24-month study follow-up. The current study examines results from the intervention group only. All study protocols were fully approved by the Harvard School of Public Health Institutional Review Board and the POWER Data Safety Monitoring Board.

### Intervention

The theoretically- and evidence-based intervention has been described in detail elsewhere [11]. Briefly, the 24-month intervention utilized skills training, 18 coaching phone calls with a community health educator, and the interactive obesity treatment approach. The interactive obesity treatment approach has been extensively tested in previous studies [12, 16, 17] and prescribes tailored, evidence-based behavior change goals in order to create a sufficient caloric deficit to produce weight change (e.g., five or more fruits and vegetables/day, no fast food, no sugar sweetened beverages, walking 10,000 steps per day). BFBW asked participants to only self-monitor simple discrete behavior goals (e.g., five or more fruits and vegetables every day) rather than detailed logs of food intake and nutrients (e.g., calories, fat, grams, etc.) to reduce burden and improve long-term engagement with self-monitoring behavior.

Community resource materials and skills training were available via the study website and in print format, with participants selecting their preferred means of receiving them. The web site and print materials had similar feel and content, although the web site allowed for interactivity. At baseline, participants were prescribed three behavioral goals and selected new goals every 13 weeks. Two goals, adhering to prescribed hypertension regimen and steps per day, remained constant throughout the intervention period. The third goal was chosen based on discussions with the community health educator.

Participants who elected for web-based materials self-monitored their behaviors on-line, while participants who opted for print materials could self-monitor their behaviors daily using a paper log and then entered this information during the weekly phone call using IVR. We designed the IVR call using response options that would be easy for the participants to complete even if they had never used the paper logs. This, for both modes, provided participants with tools to log daily. However, we expected weekly reporting for both modes. After self-monitoring data was received, participants received immediate automated tailored feedback on their progress. (e.g., *Great job with sugary drinks this past week*. *Keep it up*. *Try adding lemon to water as a drink*.) Feedback was equivalent regardless of self-monitoring modality. Participants were given the option to change to the other self-monitoring approach at any time.

### Measures

Intervention engagement was measured by frequency of weekly self-monitoring across the 24-month intervention period. Participants in the web modality were able to monitor their goals daily with an expectation that they would self-monitor at least once per week. Self-monitoring was considered complete if a minimum of one self-monitoring form was submitted to the web system each week. Participants using the IVR system were able to self-monitor daily via paper log and were classified as self-monitoring for a given week if they completed the entire weekly IVR call. Utilization of both the web and IVR system was determined by calculating the percentage of monitoring episodes completed over an approximate month (four weeks) out of the number expected (four).

We determined frequency of self-monitoring in four six-month periods (months 0–6, 7–12, 13–18, and 19–24). We also determined the number of page visits to the primary sections of the web site (skills training, action plan, recipes, and local resources) in six-month periods. As participants were able to switch from the intervention modality selected at baseline, we determined the number of individuals who opted to switch modalities.

Changes in body weight using both systolic and diastolic blood pressure were also measured using standard protocols. At baseline, participants reported their race/ethnicity, and employment status, and completed measures that assessed frequency of Internet use and Internet access across different settings (e.g., at home, at work). We also assessed health literacy level using a brief screening tool [18].

### Statistical analysis

To characterize the participants selecting each self-monitoring modality, we computed descriptive statistics for the IVR and web self-monitoring groups, as well as for those who switched modalities. Baseline categorical variables were compared between groups using chi-squared tests. Baseline continuous variables were evaluated using analyses of variance (ANOVA).

To examine the rate of change in self-monitoring over the course of the intervention, we examined monitoring rates at the week level. We assessed the effects of switching intervention modalities on self-monitoring rates by comparing the rates of participants who stayed in their original intervention modality group throughout the study to those who switched. We conducted a logistic generalized estimating equations (GEE) analysis on a dichotomous variable indicating if self-monitoring was completed for each week of the study, regardless of modality (web or IVR) and the intervention modality group (web, IVR and switchers).

The potential differential effect of socio-demographic characteristics on self-monitoring rates was examined in participants who did and did not switch modalities during the study. Between group differences in the IVR call completion rate over time were evaluated through a logistic GEE analysis (yielding odds ratios (OR)). For participants assigned to the web modality, self-monitoring events were counted for each study week after week 1. Between group differences in the rate of change in weekly self-monitoring counts over time were analyzed using a GEE approach based on a Poisson distribution with a log link function (yielding rate ratios (RR)). All GEE analyses used an autoregressive correlation structure, which was determined to be appropriate after comparison with an unstructured correlation matrix.

The associations between self-monitoring and weight loss were evaluated by grouping participants by their percentage weight loss (less than 2%, 2% to less than 5% and 5% or more) and using ANOVA to compare the total number of self-monitoring forms (web) or calls (IVR) completed. Additionally, the association between total self-monitoring in each modality and continuous measures of final weight loss, systolic and diastolic blood pressure were assessed by calculating Spearman’s rank correlation coefficients.

## Results

### Overall user engagement

The 24-month BFBW trial resulted in significantly greater reductions in weight, BMI, and blood pressure among the intervention group as compared to usual care [14]. The following data are among intervention participants only.

The majority of the 180 intervention participants selected IVR self-monitoring at baseline as compared to the web (61% vs. 39%). Web users were more likely to be employed, have some post-high school education, and to fall in the higher income categories, compared to those who selected IVR (Table 1). Web users also had higher health literacy levels and were likely to be frequent Internet users, regardless of how the Internet was accessed (at home, at work, via mobile device). Web users were significantly younger than those who selected IVR and had a higher baseline diastolic blood pressure. Only 22% of participants changed intervention modalities, all of whom switched from web to IVR (56% of participants who selected web at baseline switched to IVR, none of the participants who selected IVR at baseline changed to the web modality). Participants who switched from the web to IVR were not significantly different from those who continued to engage with the study via the web on any indicator except diastolic blood pressure (DBP). Participants who switched had a significantly higher DBP at baseline.

**Table 1:** Participant’s baseline characteristics by intervention modality (n = 180).

	IVR	Web	Switchers	p-value
	(n = 110)	(n = 31)	(n = 39)	
**Sex, N (%)**				0.42
Male	28 (25.45)	10 (32.26)	14 (35.9)	
Female	82 (74.55)	21 (67.74)	25 (64.1)	
**Race/Ethnicity, N (%)**				0.56
Non-Hispanic White	6 (5.45)	2 (6.45)	1 (2.56)	
Non-Hispanic Black/African American	76 (69.09)	22 (70.97)	31 (79.49)	
Hispanic	18 (16.36)	4 (12.9)	3 (7.69)	
American Indian	3 (2.73)	0 (0)	0 (0)	
Asian	0 (0)	0 (0)	1 (2.56)	
Hawaiian/Pacific Islander	0 (0)	0 (0)	1 (2.56)	
More than one race	7 (6.36)	3 (9.68)	2 (5.13)	
**Education N (%)**				<0.0001
< 12th grade	42 (38.18)	1 (3.23)	4 (10.26)	
High school/G.E.D.	36 (32.73)	14 (45.16)	9 (23.08)	
Some college/Associates degree	20 (18.18)	9 (29.03)	15 (38.46)	
Bachelors degree or higher	12 (10.91)	7 (22.58)	11 (28.21)	
**Income N (%)**				<0.0001
Less than $10,000	37 (33.64)	2 (6.45)	2 (5.13)	
$10,000 to less than $25,000	40 (36.36)	8 (25.81)	5 (12.82)	
$25,000 to less than $50,000	20 (18.18)	15 (48.39)	18 (46.15)	
$50,000 or more	13 (11.82)	6 (19.35)	14 (35.9)	
**Employment N (%)**				<0.0001
Employed	41 (37.27)	20 (64.52)	33 (84.62)	
Unemployed	21 (19.09)	5 (16.13)	1 (2.56)	
Retired	17 (15.45)	3 (9.68)	2 (5.13)	
Disabled	31 (28.18)	3 (9.68)	3 (7.69)	
**Healthy literacy N (%)**				<0.0001
Bottom tertile	48 (43.64)	2 (6.45)	2 (5.13)	
Middle tertile	24 (21.82)	7 (22.58)	10 (25.64)	
Top tertile	38 (34.55)	22 (70.97)	27 (69.23)	
**Internet use frequency N (%)**				<0.0001
Never	79 (71.82)	5 (16.13)	5 (12.82)	
Monthly	14 (12.73)	1 (3.23)	1 (2.56)	
Weekly	10 (9.09)	6 (19.35)	7 (17.95)	
Daily	7 (6.36)	19 (61.29)	26 (66.67)	
**Internet use location**				
** Home N (%)**				<0.0001
** **No	6 (5.45)	2 (6.45)	3 (7.69)	
** **Yes	25 (22.73)	24 (77.42)	31 (79.49)	
** **No use	79 (71.82)	5 (16.13)	5 (12.82)	
** Work N (%)**				<0.0001
** **No	23 (20.91)	14 (45.16)	15 (38.46)	
** **Yes	8 (7.27)	12 (38.71)	19 (48.72)	
** **No use	79 (71.82)	5 (16.13)	5 (12.82)	
** Mobile device N (%)**				<0.0001
** **No	29 (26.36)	26 (83.87)	31 (79.49)	
** **Yes	2 (1.82)	0 (0)	3 (7.69)	
** **No use	79 (71.82)	5 (16.13)	5 (12.82)	
** Other**				<0.0001
** **No	22 (20.00)	23 (74.19)	24 (61.54)	
** **Yes	9 (8.18)	3 (9.68)	10 (25.64)	
** **No use	79 (71.82)	5 (16.13)	5 (12.82)	
**Age (years) mean ± SD**	56.72 ± 10.76	51.87 ± 10.04	50.74 ± 10.03	0.003
**Weight (kg) mean ± SD**	98.01 ± 14.16	102.89 ± 19.60	101.93 ± 18.69	0.21
**BMI (kg/m2) mean ± SD**	37.15 ± 4.98	37.03 ± 5.3	36.73 ± 4.73	0.9
**SBP (mmHg) mean ± SD**	130.13 ± 19.00	125.53 ± 20.30	134.19 ± 16.94	0.16
**DBP (mmHg) mean ± SD**	78.38 ± 13.00	75.91 ± 10.91	84.78 ± 11.93	0.006

Note: Switchers are individuals who changed from web-based modality to IVR modality during the intervention. No participants changed from IVR to web. IVR = interactive voice response; SBP = systolic blood pressure; DBP = diastolic blood pressure; BMI = body mass index

Of the five domains of the website (administrative/home, local resources, recipes, skills tracking and self-monitoring), self-monitoring was the most frequently visited every week. Over the duration of the entire intervention, 83% of the page visits were to the self-monitoring page, followed by the administrative/home page (14%) (Table 2).

**Table 2:** Website use by 6 month period in web-only participants (n = 31) and participants who changed from web to IVR (n = 39)*.

	Months 0–6		Months 7–12		Months 13–18		Months 19–24	
	web-only (n = 31)	modality changers (n = 39)	web-only (n = 31)	modality changers (n = 18)	web-only (n = 31)	modality changers (n = 10)	web-only (n = 31)	modality changers (n = 4)
**Self-monitoring**								
Mean (SD)	80.71 (67.53)	30.79 (41.99)	55.1 (69.84)	24.06 (42.05)	40.19 (69.29)	14.8 (24.61)	32.9 (60.30)	38.5 (44.50)
Median	62	9	0	0	0	2.5	0	29.5
Range	[0, 171]	[0, 146]	[0, 177]	[0, 140]	[0, 179]	[0, 72]	[0, 175]	[0, 95]
**Action plan**	(n = 31)	(n = 39)	(n = 31)	(n = 18)	(n = 31)	(n = 9)	(n = 31)	(n = 1)
Mean (SD)	27.26 (57.52)	13.64 (29.57)	3.35 (9.05)	5.72 (14.88)	2.87 (13.51)	0.33 (1)	0.71 (2.38)	0 (-)
Median	5	1	0	0	0	0	0	0
Range	[0, 270]	[0, 142]	[0, 42]	[0, 61]	[0, 75]	[0, 3]	[0, 10]	[0, 0]
**Recipes**	(n = 31)	(n = 39)	(n = 31)	(n = 18)	(n = 31)	(n = 9)	(n = 31)	(n = 1)
Mean (SD)	27.1 (42.10)	9.49 (13.99)	5.39 (15.18)	3.33 (6.78)	4.35 (17.67)	0.33 (0.71)	0.9 (2.65)	0 (-)
Median	15	5	0	0	0	0	0	0
Range	[0, 222]	[0, 79]	[0, 77]	[0, 24]	[0, 97]	[0, 2]	[0, 12]	[0, 0]
**Skills training**	(n = 31)	(n = 39)	(n = 31)	(n = 18)	(n = 31)	(n = 9)	(n = 31)	(n = 1)
Mean (SD)	45.1 (66.45)	18.31 (29.85)	4.74 (11.56)	6.83 (20.08)	4.23 (16.40)	1.89 (2.89)	1.94 (7.64)	0 (-)
Median	18	3	0	0	0	0	0	0
Range	[0, 292]	[0, 156]	[0, 52]	[0, 84]	[0, 90]	[0, 7]	[0, 41]	[0, 0]
**Local Resources**	(n = 31)	(n = 39)	(n = 31)	(n = 18)	(n = 31)	(n = 9)	(n = 31)	(n = 1)
Mean (SD)	5.48 (8.70)	2.82 (5.11)	0.65 (1.98)	1.17 (2.98)	0.03 (0.18)	0.11 (0.33)	0.55 (1.80)	0 (-)
Median	1	1	0	0	0	0	0	0
Range	[0, 28]	[0, 25]	[0, 9]	[0, 11]	[0, 1]	[0, 1]	[0, 9]	[0, 0]

*Website use is measured by page views except for self-monitoring where it is measured by completion of the self-monitoring form.

### Self-monitoring engagement

**Fig 1:**
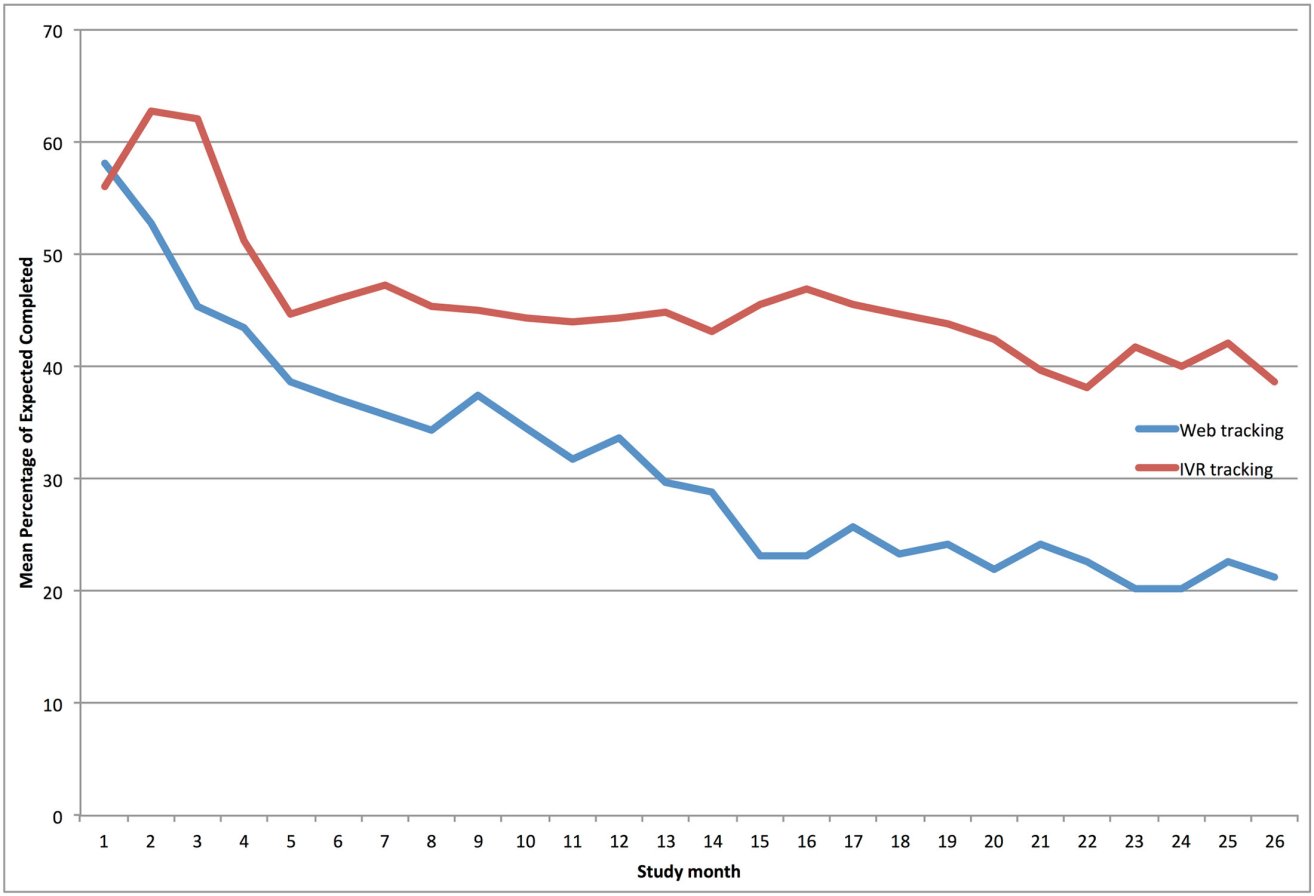
Mean percentage of self-monitoring completion over a four-week period in Be Fit Be Well.

Among IVR users, the average percentage of web-monitoring completed in the first month was 56%, which rose to 63% in month two. This declined to 46% by the sixth month and was 39% by the final month (Fig 1). Among web users, 58% completed self-monitoring logs, but that percentage declined to an average of 37% by the sixth four week period and was 22% by the final four week period. Among those who did not change self-monitoring modalities, the decline in self-monitoring was greater among those who used web-monitoring throughout the intervention than among those who used IVR (p = 0.0013).

In the first six months, participants who remained in the web-based modality (n = 31) completed the form an average of 78.0 times (Table 2). This declined to 32.9 times by the final six months of the intervention. In contrast, participants who switched from the web to IVR (the timing of the switching varied by individual), completed the self-monitoring form an average of 29.5 times in the first six months, 14.8 times in the first half of year two and 38.5 times in the last six months of the intervention.

### Engagement trends by sociodemographic characteristics

For patients who opted for IVR self-monitoring, few variables were significantly associated with engagement over time (Table 3). Participants with high health literacy levels were more likely to decrease IVR self-monitoring as compared to those with the lowest health literacy (p = 0.04). Participants who used mobile phones to access the Internet had a significantly more rapid decrease in self-monitoring than those who did not (p<0.0001).

**Table 3:** Association between patient characteristics and change in self-monitoring rate by self-monitoring modality over a 13 week period.

	Odds ratio [95%CI]	P-value	Rate ratio [95%CI]	P-value
**Sex**		0.98		0.32
Male	ref		ref	
Female	1.002 [0.90, 1.11]	0.98	1.12 [0.89, 1.41]	0.32
**Race/Ethnicity**		0.25		0.02
Non-Hispanic Black/African American	ref		ref	
Hispanic	1.07 [0.93, 1.23]	0.35	0.71 [0.56, 0.90]	4
Other	0.92 [0.80,1.05]	0.22	0.95 [0.76, 1.18]	0.64
**Education**		0.32		0.08
< 12th grade	ref		ref	
High school/G.E.D.	0.90 [0.81, 1.01]	0.07	1.03 [0.84, 1.27]	0.78
Some college/Associates degree	0.96 [0.85, 1.09]	0.57	1.05 [0.90, 1.24]	0.53
Bachelors degree or higher	0.93 [0.78, 1.11]	0.43	1.15 [1.03, 1.27]	0.01
**Income** **		0.80		
Less than $10,000	ref			
$10,000 to less than $25,000	1.04 [0.92, 1.18]	0.50		
$25,000 to less than $50,000	0.98 [0.86,1.13]	0.83		
$50,000 or more	0.98 [0.85, 1.14]	0.82		
**Income** **				0.03
Less than $25,000			ref	
$25,000 or more			1.28 [1.03, 1.59]	0.03
**Employment**		0.57		<.0001
Employed	ref		ref	
Unemployed	0.99 [0.88, 1.12]	0.9	1.07 [0.91,1.25]	0.44
Retired	0.95 [0.80, 1.12]	0.52	0.91 [0.83, 1.003]	0.06
Disabled	0.93 [0.82, 1.04]	0.21	0.67 [0.57, 0.80]	<.0001
**Healthy literacy tertile**		0.13		0.18
Bottom tertile	ref		ref	
Middle tertile	0.99 [0.85, 1.12]	0.76	0.999 [0.84, 1.19]	0.997
Top tertile	0.90 [0.81,0.997]	0.04	1.1 [0.99, 1.23]	0.07
**Internet use frequency**		0.68		4
Never	ref		ref	
Monthly	0.98 [0.86, 1.11]	0.77	1.45 [1.15, 1.83]	2
Weekly	1.08 [0.91, 1.29]	0.37	1.19 [0.87, 1.63]	0.27
Daily	0.95 [0.80, 1.11]	0.51	1.28 [0.99, 1.66]	0.06
**Internet use location**				
** Home**		0.75		0.01
** **No	ref		ref	
** **Yes	0.91 [0.72, 1.16]	0.45	0.91 [0.81, 1.03]	0.14
** **No use	0.92 [0.74, 1.16]	0.49	0.72 [0.57, 0.91]	0.01
** Work**		0.92		4
** **No	ref		ref	
** **Yes	0.96 [0.75, 1.22]	0.72	1.19 [1.03,1.39]	0.03
** **No use	0.99 [0.89, 1.09]	0.78	0.85 [0.65, 1.11]	0.23
** Mobile device**		<.0001		0.05
** **No	ref		ref	
** **Yes	0.14 [0.09, 0.23]	<.0001	No participants	
** **No use	0.99 [0.89, 1.10]	0.88	0.78 [0.61, 0.998]	0.05
** Other**		0.99		0.31
** **No	ref		ref	
** **Yes	0.99 [0.86, 1.14]	0.87	1.09 [0.95, 1.25]	0.23
** **No use	0.99 [0.87, 1.12]	0.88	0.79 [0.61,1.06]	0.08
**Age (yr)**	0.999 [0.99, 1.004]	0.74	1.003 [0.997, 1.009]	0.37
**Weight (kg)**	0.999 [0.99, 1.003]	0.77	0.996 [0.99, 0.999]	0.01
**BMI (kg/m2)**	0.99 [0.99, 1.01]	0.68	0.99 [0.98, 1.002]	0.1
**SBP (mmHg)**	0.999 [0.999, 1.002]	0.77	0.9999 [0.997,1.003]	0.93
**DBP (mmHg)**	1.001 [0.99, 1.005]	0.64	0.996 [0.99, 1.005]	0.43

BMI: body mass index; SBP: systolic blood pressure, DBP: diastolic blood pressure

*web-based users were those to remained in the web-based modality for the duration of the period

** due to small numbers, groups were not included or combined for web-based modality

For participants using the web page to self-monitor, higher health literacy (p = 0.07), having a bachelor’s degree (p = 0.01), income above $25,000 (p = 0.03) and more frequent Internet use (p = 0.004) were associated with a less rapid decline in rates of self-monitoring. Hispanic ethnicity (vs. non-Hispanic/Black, p = 0.004) and being retired (p = 0.06) or disabled (p<0.001) were associated with a more rapid decline in self-monitoring. Baseline weight was also inversely associated with web-based self-monitoring (p = 0.01); those with a higher weight were on average more likely to decrease frequency of web-based monitoring. Compared to participants who use the Internet at home, those who did not saw a greater decline in web-based self-monitoring (p = 0.01). In contrast, participants who use the Internet at work for personal matters were more likely to continue self-monitoring over time as compared to those who did not use the Internet at work (p = 0.03).

### Self-monitoring and physiological outcomes

As is shown in Table 4, there was no association between self-monitoring and average percent weight loss for either modality. Though not significant, there was a trend of greater weight loss with increasing self-monitoring. Participants in the highest quartile of web self-monitoring lost 4.21 kg (sd = 10.75 kg) while those in the lowest quartile were essentially unchanged ($\bar{x}$ = 0.18 kg, sd = 4.05 kg). Similarly, while those in the lowest quartile of IVR self-monitoring frequency were weight stable ($\bar{x}$ = 0.12 kg, sd = 4.07 kg), those in the highest quartile of self-monitoring lost nearly 2 kg ($\bar{x}$ = 1.51 kg, sd = 6.25 kg).

**Table 4:** Correlations between self-monitoring and weight and blood pressure outcomes.

	n	mean	sd	median	IQR	Spearman's Rho	P-value
**IVR tracking**	110	47.62	37.02	48.50	[10.00, 85.00]	ref	ref
Weight loss (kg)	110	1.36	5.75	0.58	[-1.20, 4.10]	0.13	0.18
Weight loss (%)	110	1.33	5.45	0.54	[-1.40, 4.19]	0.11	0.23
SBP increase (mmHg)	110	2.60	19.84	0	[-8.00, 11.34]	0.004	0.96
DBP increase (mmHg)	110	0.49	12.92	0	[-7.00, 8.00]	0.03	0.75
**Web tracking**	31	206.22	241.43	57	[8.00, 309.00]	ref	ref
Weight loss (kg)	31	2.14	6.54	0	[-0.89, 4.60]	0.11	0.56
Weight loss (%)	31	1.95	6.25	0	[-0.80, 4.34]	0.13	0.48
SBP increase (mmHg)	31	1.09	18.70	0	[-9.60, 11.00]	-0.06	0.75
DBP increase (mmHg)	31	1.97	11.23	2.34	[-3.00, 5.40]	0.13	0.49

Improvements in blood pressure were not associated with self-monitoring engagement (Table 4). For participants who chose web-based tracking, the correlation between self-monitoring and both systolic (r = -0.06, p = 0.75) and diastolic (r = 0.13, p = 0.49) blood pressure was small and non-significant. For participants who chose IVR-based tracking, results were similar for systolic (r = 0.004, p = 0.96) and diastolic (r = 0.03, p = 0.75) blood pressure.

## Discussion

This is the first study to compare the different eHealth-based self-monitoring modes, Web and Interactive Voice Response (IVR), in a weight-loss intervention in which participants self-selected their self-monitoring modality. Regardless of modality selected, self-monitoring declined over time, as has been seen previously with both in-person and other eHealth interventions [7]. This is also one of the few studies to examine long-term (greater than six months) engagement in various eHealth self-monitoring tools. Participants who selected IVR based monitoring were more likely to be compliant from the third month on. This is important as many commercial eHealth tools fail to monitor engagement past 12 weeks. We were able to maintain high levels of self-monitoring in the IVR modality (over 40% of participants engaged at least once a week at one year and this held fairly constant in the subsequent 12 months).

Similar to previous studies, we saw a decline in engagement over time. However, the magnitude of decline, in the IVR arm in particular, was markedly less than expected based on previous studies testing eHealth approaches to self-monitoring in this patient population. After an initial spike in use of IVR, which may be attributed to the novelty of the technology, engagement with the intervention was durable, declining from 46% in the first month to 39% at the end of the study. In contrast, web-based self-monitoring followed a declining pattern more similar to those previously reported [6, 9].

The reasons why engagement was higher with the IVR modality are a key area for future research. Whether participants preferred the phone-based approach for self-monitoring or that it was simpler, quicker, more portable or didn’t require them to get on the computer, or any number of other differences between approaches may help focus future efforts on IVR phone, text or app-based approaches to self-monitoring and feedback [19, 20]. The BFBW web site was not mobile optimized and was not available as an app, as was typical at the time of the study. It is possible that despite designing for a low literacy audience our web-modality was not as accommodating as we hoped and participants experienced challenges with using that platform. It is also possible that contextual barriers (e.g., workplace limitations on use of computers among those without home service, slow or unstable service at home) may have contributed to lower usage in this group [21]. Previous studies have shown that mobile phone based self-monitoring may improve adherence [12, 17, 22].

Access to Internet at home or work may have increased the likelihood of continuing to engage in web-based self-monitoring and suggests that easy access to self-monitoring tools is an important consideration in developing future tools/interventions. The importance of easy access to self-monitoring tools is further supported by the higher frequency of switching from web to IVR than from IVR to web. Our intervention did not specifically evaluate self-monitoring via web-enabled phones, as the study largely predated use of smartphones, and future investigations might compare IVR to web app or text-based monitoring via phones since these modalities would be easily accessed regardless of location. Given the high use of smartphones among lower socioeconomic status populations [23, 24] this is a question worthy of future attention. While self-monitoring was the most frequent reason for using the website, participants also engaged, particularly in the early weeks of the study, with skills training and action-planning. These data suggest that eHealth interventions focused solely on self-monitoring are missing a significant need among this population that may be important for driving continued engagement, sustained behavior change and improved health outcomes.

Although all participants were overall low socioeconomic position, web users who were more socioeconomically advantaged experienced slower declines in self-monitoring that those who were lower socioeconomic status, which also lends support to the notion that contextual and access variables are important in achieving compliance with self-monitoring. It suggests that successful eHealth interventions must do more than simply provide self-monitoring tools and find ways to promote and facilitate self-monitoring in these hard to reach populations. Some recent studies have tested text messaging for tracking behavioral goals and have shown promising results for enhancing self-monitoring adherence among more socioeconomically disadvantaged populations [12, 25].

Despite the success of the BFBW intervention at decreasing weight and blood pressure relative to control using web and IVR based self-monitoring tools and the robust literature on the importance of self-monitoring to support behavior change, engagement with self-monitoring tools did not predict change in weight loss or blood pressure. Given the focus on self-monitoring tools, particularly in the commercial eHealth market, a better understanding of how to optimize self-monitoring (e.g., through improved user interface or more frequent prompts) to improve outcomes may be necessary to achieve larger effects.

As researchers and practitioners increasingly look for more cost effective ways of reaching large numbers of patients and delivering healthy lifestyle interventions, data on whether the degree of engagement with the intervention is associated with outcomes is important for resource allocation and future study planning. Our study provides important information about engagement with eHealth interventions for high-risk, populations, which can inform the development of future interventions for these populations. In particular, attention to providing the option to change self-monitoring modalities is an important element to enhancing intervention engagement.
